# Efficacy of pegylated interferon plus ribavirin in combination with corticosteroid for two cases of combined hepatitis C and autoimmune hepatitis

**DOI:** 10.1007/s12328-012-0295-4

**Published:** 2012-03-28

**Authors:** Satoshi Oeda, Toshihiko Mizuta, Hiroshi Isoda, Takuya Kuwashiro, Noriko Oza, Shinji Iwane, Hirokazu Takahashi, Yasunori Kawaguchi, Yuichiro Eguchi, Shuji Toda, Iwata Ozaki, Keizo Anzai, Kazuma Fujimoto

**Affiliations:** 1 Department of Internal Medicine, Saga Medical School, 5-1-1 Nabeshima, Saga, 849-8501 Japan; 2 Department of Pathology, Saga Medical School, 5-1-1 Nabeshima, Saga, 849-8501 Japan

**Keywords:** Autoimmune hepatitis, Chronic hepatitis C, Interferon, Ribavirin, Corticosteroid

## Abstract

The treatment strategy for cases of combined autoimmune hepatitis (AIH) and chronic hepatitis C (CHC) has not yet been established. A 47-year-old woman and a 53-year-old-woman were hospitalized for treatment of CHC. Ultrasonography and histological findings revealed that their liver was not cirrhotic but did have chronic damage. The histological findings of both patients were suggestive of AIH. The patients were systematically treated with pegylated interferon-alpha 2b plus ribavirin which was preceded by and combined with corticosteroid (CS), and showed sustained virological responses and normal liver function. Although these two patients with combined AIH and CHC were successfully treated with this regimen, careful attention to exacerbation of hepatic inflammation is needed because hepatitis C viral load was increased due to immunosuppression during CS treatment.

## Introduction

Hepatitis C virus (HCV) infection is known to be associated with various autoimmune diseases, such as autoimmune hepatitis (AIH), Sjögren’s syndrome, rheumatoid arthritis and autoimmune thyroid disorders [1]. Among AIH patients, it has been reported that at least 10 % were infected with HCV in Japan [2]. Although corticosteroid (CS) therapy has been established as effective for AIH [3–5], there is concern about the possible increase in HCV caused by the immunosuppressive effect of CS in HCV-infected AIH patients. In contrast, interferon (IFN) administration, which is effective for chronic hepatitis C (CHC), has been reported to initiate acute exacerbation of AIH [6], and sometimes fulminant hepatic failure [7, 8]. Owing to these discordant treatment options for AIH and CHC, the treatment decision for patients with both of these hepatic diseases represents a dilemma.

Here we report two patients with combined AIH and CHC who showed favorable outcomes with pegylated IFN (PEG-IFN) plus ribavirin (RBV) therapy which was preceded by and combined with CS administration.

## Case reports

### Case 1

A 47-year-old woman (height 153.3 cm, weight 64.5 kg) was referred to our hospital for treatment of CHC in August 2006. Although she had received IFN therapy 5 years previously, eradication of HCV had not been achieved, and her serum levels of transaminases during the therapy had been higher than baseline.

**Fig. 1:**
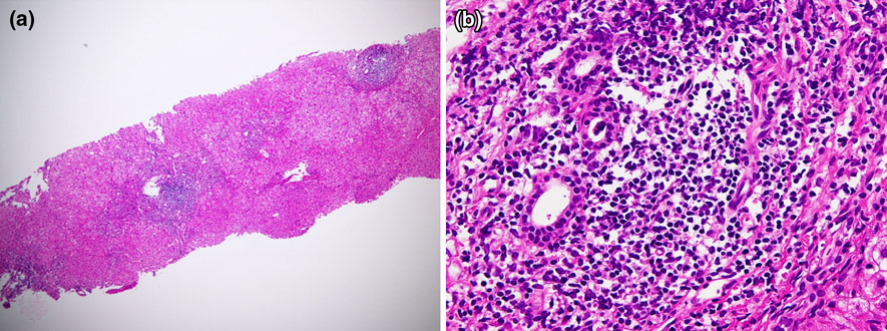
Histological findings of Case 1 show considerable infiltration of plasma cells in portal areas and severe interface hepatitis. **a** H&E ×40, **b** H&E ×400

She was not a habitual drinker, and there was no history of blood transfusion, drug abuse or tattoos. There were no abnormal findings in her physical examination. Blood tests showed that the alanine aminotransferase (ALT) level was 97 IU/L, immunoglobulin (Ig) G concentration was 3457 mg/dL, anti-nuclear antibody (ANA) titer was 1:40, liver–kidney microsomal antibody-1 (LKM-1) was negative, HCV genotype was 2a and viral load was 2700 KIU/mL (Table 1). HLA typing showed DR4. To distinguish between AIH and CHC, a liver biopsy was carried out under laparoscopy. Although characteristic laparoscopic findings for AIH of multilobular and ecchymotic red macula, extensive recess, furrowed recess and rough and large tuber were not seen; microscopic findings showed considerable infiltration of plasma cells in portal areas and severe interface hepatitis, which are uncommon in CHC (Fig. 1). Therefore, we determined that the main cause of her hepatic disorder was AIH, although the diagnostic score according to the International Autoimmune Hepatitis Group (IAIHG) in 1999 [9] was 12 points, defined as ‘probable’ for AIH.

**Table 1:** Laboratory data on admission (Case 1)

WBC	4600/μL	Total protein	8.2 g/dL	IgG	3457 mg/dL
RBC	434 × 10^4^/μL	Albumin	3.8 g/dL	IgA	299 mg/dL
Hb	13.1 g/dL	*γ*-globulin	31.9 %	IgM	47 mg/dL
Ht	39.4 %	AST	82 IU/L	ANA	40 times
Platelet	14.7 × 10^4^/μL	ALT	97 IU/L	ASMA	(−)
		LDH	226 IU/L	LKM-1 Ab	(−)
		ALP	225 IU/L	AMA	(−)
		γGTP	48 IU/L	HBs Ag	(−)
		Total bilirubin	0.7 mg/dL	HBc Ab	(−)
		Cholinesterase	266 IU/L	HCV-RNA	2700 KIU/mL
				Genotype	2a
				HLA-DR	4, 9

*ANA* anti-nuclear antibody, *ASMA* anti-smooth muscle antibody, *LKM-1 Ab* liver−kidney microsomal antibodies type 1, *AMA* antimitochondrial antibody

**Fig. 2:**
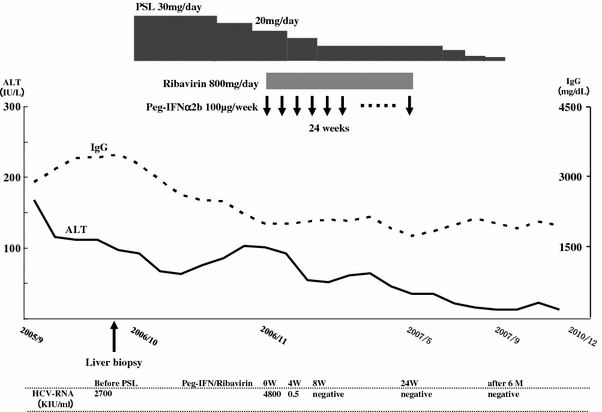
Clinical course of Case 1

We started 30 mg/day prednisolone (PSL) administration in October 2006. Although the IgG level gradually decreased after initiation of PSL, the ALT level remained unchanged. Serum HCV load increased to 4800 KIU/mL during PSL administration. After 6 weeks of PSL (ALT 101 IU/L, IgG 2008 mg/dL), a weekly subcutaneous injection of 100 μg PEG-IFN-alpha-2b and daily oral administration of 800 mg RBV were started in combination with 20 mg/day PSL. The ALT level decreased gradually after starting PEG-IFN plus RBV therapy, and HCV RNA disappeared from her serum at week 8 of PEG-IFN plus RBV therapy. Subsequently, a sustained virological response (SVR) was achieved by PEG-IFN plus RBV therapy for 24 weeks. PSL was continued for 4 months after cessation of PEG-IFN plus RBV, and then withdrawn because ALT and IgG levels remained continuously normal (ALT 13 IU/L, IgG 1472 mg/dL at the end of PSL administration). From the end of the treatment to the present time, her serum ALT and IgG levels have been within the normal ranges for 3 years without any medication (Fig. 2).

### Case 2

Case 2 was a 53-year-old woman (height 159 cm, weight 56.6 kg). She was diagnosed with CHC at 39 years of age, but had not taken any medication because of low serum ALT levels. Symptoms of dry eye and mouth appeared in January 2008, and she was diagnosed with Sjögren’s syndrome and mixed connective tissue disease based on the symptoms and serological tests, by a specialist in collagen diseases. Her hepatic function worsened after oral administration of pilocarpine hydrochloride, therefore, she was referred to our department.

**Fig. 3:**
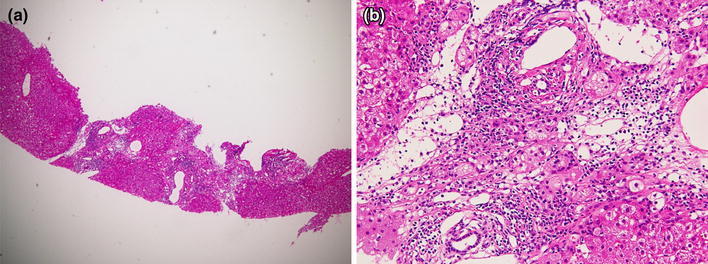
Histological findings of Case 2 show moderate infiltration of lymphocytes and plasma cells in portal areas, interface hepatitis and rosette formation. **a** H&E ×40, **b** H&E ×400

She had a history of transfusion of blood coagulation factors during childbirth. Laboratory tests showed that theALT level was 128 IU/L, IgG level was 1933 mg/dL, ANA titer was 1:1280, LKM-1 was negative, HLA typing showed DR9 and DR15, HCV genotype was 1b and viral load was 3.3 log IU/mL (Table 2). Histological findings of a liver biopsy specimen showed moderate infiltration of lymphocytes and plasma cells in portal areas, interface hepatitis and rosette formation, which are typical AIH characteristics (Fig. 3). Although the score according to the simplified criteria of AIH (IAIHG 2008) [10] was 6 points, which means ‘probable’ for AIH, we judged that her hepatic disorder was mainly caused by AIH, similar to Case 1.

**Table 2:** Laboratory data on admission (Case 2)

WBC	3600/μL	TP	7.3 g/dL	IgG	1933 mg/dL	
RBC	463 × 10^4^/μL	Albumin	3.9 g/dL	IgA	322 mg/dL	
Hb	13.5 g/dL	γ-globulin	25.5 %	IgM	156 mg/dL	
Ht	40.4 %	AST	108 IU/L	ANA	1280 times	
Platelet	15.1 × 10^4^/μL	ALT	128 IU/L	ASMA	(−)	
		LDH	269 IU/L	LKM-1 Ab	(−)	
		ALP	169 IU/L	AMA	(−)	
		γGTP	85 IU/L	HBs Ag	(−)	
		Total bilirubin	1.3 mg/dL	HBc Ab	(+)	
		Cholinesterase	290 IU/L	HBV-DNA	(−)	
				HCV-RNA	3.3 log IU/mL	
				Genotype	1b	
				HLA-DR	9, 15	

*ANA* anti-nuclear antibody, *ASMA* anti-smooth muscle antibody, *LKM-1 Ab* liver−kidney microsomal antibodies type 1, *AMA* antimitochondrial antibody

**Fig. 4:**
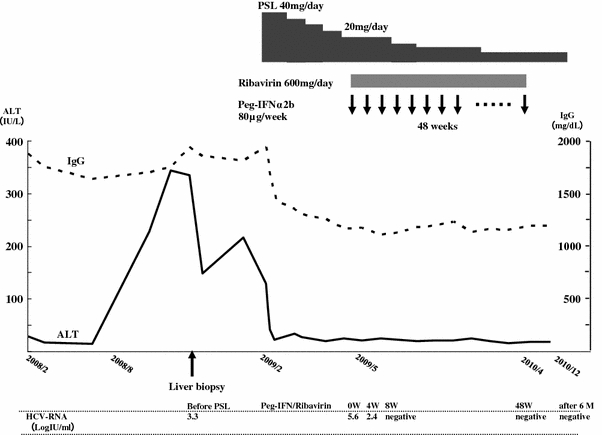
Clinical course of Case 2

After starting oral administration of 40 mg PSL (0.7 mg/kg) in February 2009, her ALT and IgG levels immediately decreased and became normalized. Serum HCV load increased to 5.6 log IU/mL during PSL administration. After PSL administration for 13 weeks, with a gradual decrease in dose, a weekly subcutaneous injection of 80 μg PEG-IFN-alpha-2b and daily oral 600 mg RBV were started in combination with 20 mg/day PSL. HCV RNA disappeared from her serum at week 8 of PEG-IFN plus RBV therapy, and an SVR was achieved by continuing the treatment for 48 weeks. After the end of the PEG-IFN plus RBV therapy, PSL dose was gradually decreased and daily administration of 5 mg has continued to date. Consequently, her ALT and IgG levels have remained within the normal range (Fig. 4).

## Discussion

We have reported two patients with features of AIH together with HCV infection, who were successfully treated with PEG-IFN plus RBV therapy preceded by and combined with steroid administration. This therapeutic challenge may represent one approach for hepatitis in patients with combined AIH and CHC.

The most important issue in this approach is how to judge whether the autoimmunity is associated with hepatic inflammation in patients with HCV infection.

CHC patients sometimes become positive for autoantibodies such as ANA, therefore, it is difficult to distinguish serologically between simple CHC and CHC combined with AIH. A variety of type 2 AIH, which is characterized by anti-LKM-1 antibodies in the serum, has been reported with HCV-associated AIH [11]. However, the positivity rate of anti-LKM-1 antibodies in Japanese CHC patients is low [12], and our two cases were actually both negative.

There are some reports indicating the importance of histological manifestations such as severe piecemeal necrosis, lobular hepatitis, multinucleated giant cells, and moderate or severe infiltration of plasma cells, which are microscopic characteristics of AIH, to distinguish CHC accompanied with AIH from simple CHC [13].

We diagnosed CHC combined with AIH based on changes in the level of ALT during previous IFN treatment and histological findings in Case 1, and on other accompanying autoimmune diseases and histological findings in Case 2.

However, the treatment strategy for combined AIH/HCV has not yet been established. It is known that IFN often induces acute exacerbation of AIH, and occasionally fulminant hepatic failure [6–8], therefore, many reports have recommended CS therapy for these patients [14, 15]. In contrast, there are some reports showing that IFN therapy is more effective than CS, even in combined AIH/CHC [16]. Petersen-Benz et al. [17] reported successful treatment of a case with AIH/CHC overlap syndrome. First, they treated AIH with CS for several years, and then switched to IFN plus RBV therapy for CHC, and achieved HCV eradication. However, readministration of CS was required to inhibit hepatic inflammation in this case. Therefore, we planned pretreatment with CS and subsequent IFN plus RBV therapy combined with continued CS for our two cases. PEG-IFN plus RBV therapy was started at 6 weeks of CS treatment in Case 1 versus at 13 weeks in Case 2 because the ALT level did not decrease steadily with CS administration in Case 1. As a result, favorable viral eradication was achieved without aggravation of hepatic inflammation in both cases.

Careful attention to viral breakthrough caused by the immunosuppressive effect of CS is required. Indeed, HCV load increased during CS administration in both of our cases. Therefore, when this therapeutic regimen is administered, it is necessary to monitor the ALT level closely until start of antiviral therapy, so as not to miss any exacerbation of hepatic inflammation.

Judging from the changes in IgG and ALT levels during CS treatment, it is speculated that the hepatic inflammation in Case 1 was caused by both HCV and autoimmunity, whereas that in Case 2 was mainly caused by AIH. Therefore, after termination of IFN plus RBV therapy, we attempted to stop CS treatment in Case 1, but continued a low dose PSL in Case 2.

In conclusion, although antiviral therapy combined with CS needs to be carefully applied, it may represent a worthwhile treatment for CHC patients with clinical and histological characteristics of AIH.
